# Experimental Research on the Behavior of the Rail Seat Section of Different Types of Prestressed Concrete Sleepers

**DOI:** 10.3390/ma13112432

**Published:** 2020-05-26

**Authors:** Aidas Jokūbaitis, Gediminas Marčiukaitis, Juozas Valivonis

**Affiliations:** Department of Reinforced Concrete Structures and Geotechnics, Faculty of Civil Engineering, Vilnius Gediminas Technical University, Saulėtekio av. 11, LT-10223 Vilnius, Lithuania; jonas.gediminas.marciukaitis@vgtu.lt (G.M.); juozas.valivonis@vgtu.lt (J.V.)

**Keywords:** prestressed concrete, railway sleepers, crack width, dynamic load, rail seat

## Abstract

Nowadays, prestressed concrete sleepers are the most common type of sleepers. In different countries all over the world prestressed concrete sleepers are designed with different shapes, with different types of prestressed reinforcement and arrangements in cross-section. Additionally, different manufacturing methods and techniques are used for prestressed concrete sleepers. These railway members are stiff, durable and can withstand heavy loads. Despite that, damaged or deteriorated prestressed concrete sleepers appear on railway tracks even before the end of their service life. Therefore, there is a need for better understanding of the behavior of different types of prestressed concrete sleepers to optimize their design. The rail seat section of the sleeper is the most affected part of sleeper. Therefore, analysis of experimental results of bearing capacity, cracking, and deformation of the rail seat section of sleeper under static and dynamic loads are provided in this article. Furthermore, different types, diameters, and anchoring methods of prestressed reinforcement were analyzed in this article. Additionally, comparison of experimental results of different types of prestressed concrete sleepers is discussed.

## 1. Introduction

The railway track has the purpose to transfer adequately the trainload to the structure of railway track. During this transfer, it has to be ensured that the track components are not subjected to loads beyond their bearing capacity. Railway track consists of two main parts: superstructure and substructure. The superstructure consists of rails, rail pads, sleepers, and fastening systems while the substructure consists of ballast, sub-ballast, and subgrade. Each layer of the railway structure reduces the effect of the load on the underlying layer and thus distributes the load evenly between the layers. Furthermore, connecting the superstructure and substructure of the track, sleepers are the most affected part of the railway structure. They withstand static, cyclic, and impact loads of different types, directions and sizes caused by trains, and are affected by support reactions of the ballast [1,2,3,4]. Additionally, sleepers are affected by environmental influences (frost, humidity, temperature variation, aggressive materials) [5] and production processes [5,6]. Furthermore, sleepers need to ensure adequate distance between the rails; evenly distribute loads from the rails to the ballast; maintain adequate inclination of the rails; act as support for the rail; restrict longitudinal, vertical, and horizontal rail displacement; and be resistant to wear. All of these factors, individually or together, affect the sleeper throughout its life cycle and can lead to damage of sleeper. Therefore, sleepers influence the global behavior of the track. Additionally, the behavior of sleepers depends on the characteristics of other track components, especially rail pads and ballast properties.

Ballast in railway track performs an important function to reduce the stress to a level which the structure of the railway track can safely withstand without any unacceptable settlement. Additionally, it provides a suitable foundation for the sleepers and also holds the sleepers in their correct level and position, preventing lateral or longitudinal displacement. The performance of a sleeper to withstand lateral and longitudinal load is dependent upon the sleeper’s size, shape, surface geometry, weight, and spacing.

The rail pad is one of the less visible parts of the fastening system. However, it has an essential function in damping the effect of vertical loading (particularly impact loading). This has two aspects: by providing an adjusting layer between the rail and the sleeper, the pad ensures even pressure on the rail seat area and by acting as a spring the pad reduces the transmission of vibration and impact from the rail into the sleeper.

Historically, timber, steel, and concrete sleepers were introduced in different countries taking into account climatic conditions and accessibility of local materials. However, nowadays new types of material are under consideration of using it in railway sleepers for a specific application or to obtain desired properties eliminating disadvantages of conventionality used timber, steel, or concrete sleepers. Composite sleepers are introduced to withstand harsh environmental impacts and eliminate damage of reinforcement due to corrosion of steel [7]. High strength concrete instead of normal concrete can be used to increase sleeper resistance to cracking and bearing capacity [8]. However, these types of sleepers have some disadvantages, have not yet been widely adopted, and are still under investigation.

The comparison of the behavior of timber, steel, and concrete sleepers showed that prestressed concrete sleepers have higher bearing capacity than timber and steel sleepers. Additionally, the deflection and ductility of prestressed concrete sleepers are lower and it provides more uniform load distribution under the sleeper compared to timber and steel sleepers [9]. Therefore, nowadays, prestressed concrete sleepers are the most widely used sleepers in the world. The advantages of prestressed concrete sleepers such as rigidity, durability, improved geometric retention of track, and greater weight vital for high-speed and heavy freight lines, higher bearing capacity, resistance to environmental impacts and cracking, low cost of maintenance makes it the best choice for the heavily loaded railway tracks.

Many different types of sleepers have been designed and manufactured in recent decades. The quality of prestressed concrete sleepers is very important for the safety of the railway. Firstly, the quality of concrete sleepers is very dependent on the quality of manufacturing process. Two main manufacturing methods are currently used in the world that is a long line method and a single mold method [6,10]. In longline method 100–200 m long formwork tables are used which has 4–8 parallel sleeper molds. This method is based on pre-tensioning steel wires or strands to abutments before concrete is poured. After concrete reaches required strength the reinforcement is released and pretensioned force is transferred to concrete through bond between reinforcement and concrete. Therefore, utilization of pretension force, stiffness, and behavior of prestressed concrete sleeper are very dependent on the quality and strength of bond between reinforcement and concrete. In turn, bond of prestressed reinforcement is very dependent on surface roughness (indentations, helical shape, etc.) of reinforcement, concrete quality, and strength and Hoyer (wedge) effect at the end of the sleeper.

Single mold method uses one sleeper long molds with up to four parallel sleepers. As in the case of longline method, this method is based on pretensioning steel bars to molds before concrete is poured. Additionally, pretensioned bars are attached to the steel bearing plates by cold-formed buttons at their tips at each end of the mold. Therefore, the prestressing force is introduced to concrete through the steel bearing plates at the ends of the sleeper and bond between reinforcement and concrete. Usually, plain bars are used in this type of manufacturing method. Therefore, the bond portion is small. However, the bond can be increased by introducing indented bars.

The behavior and stiffness of the sleeper are very dependent on the ability to transfer prestressing force to concrete. Depending on the method of manufacturing of the sleepers the transfer of prestressing force to concrete can be ensured only through bond between reinforcement and concrete [11,12] (long line method) or through bearing steel plates at the ends of the sleeper and bond (single mold method). Therefore, it is very important to meet requirements for transfer length during the release of reinforcement and anchorage length in service [13] in case of long line manufacturing method. However, the design of prestressed concrete sleepers leads to short distance between the center of the rail seat section and end of the sleeper. It means that smaller diameter reinforcement due to shorter transfer and anchorage length is used in prestressed concrete sleepers [4,5,14,15,16,17].

Monoblock prestressed concrete sleepers are used extensively throughout the world and are in use for all line types including high-speed lines and heavy haul lines. Despite the improvement in the quality of materials for sleepers, some sleepers are damaged in service and do not withstand expected service life [1,4,18]. Hence, the sleeper design becomes a core factor in track safety. Additionally, the increasing traffic and heavier loads in railways raise the need for optimizing the design of prestressed concrete sleepers [19].

Data related to the comparison of different prestressed concrete sleepers is very important for the designing and manufacturing stage and service stage of the sleepers. Some studies were performed [1,4,5,14] comparing unexploited sleepers with damaged sleepers taken from the railway to determine the cause of deterioration of the sleeper. Other rstudies were conducted to compare the behavior of sleepers made out of different materials (concrete, timber, steel) [9]. However, the behavior of prestressed concrete sleepers is complex not only due to various impacts affecting them in service but also due to different design approaches and manufacturing methods. As there are various types of monoblock prestressed concrete sleepers in Europe, there is a need for better understanding its behavior. However, there is a lack of qualitative data comparing the behavior of different types of sleepers. Therefore, this article presents a detailed comparative analysis of three types of sleepers under the influence of static and dynamic load. As the rail seat section is the most severely affected part of sleeper the influence of different diameter and type (strands, indented, and plain bars) of prestressed reinforcement were analyzed on the behavior of rail seat section of prestressed concrete sleepers. Tested sleepers were manufactured according to different methods (long line method and single mold method) and different reinforcement prestressing techniques were used. Therefore, the influence of different types of anchoring methods of prestressed reinforcement was also analyzed on the behavior of rail seat section of different types of prestressed concrete sleepers.

## 2. Materials and Methods

### 2.1. Specimens

**Figure 1:**
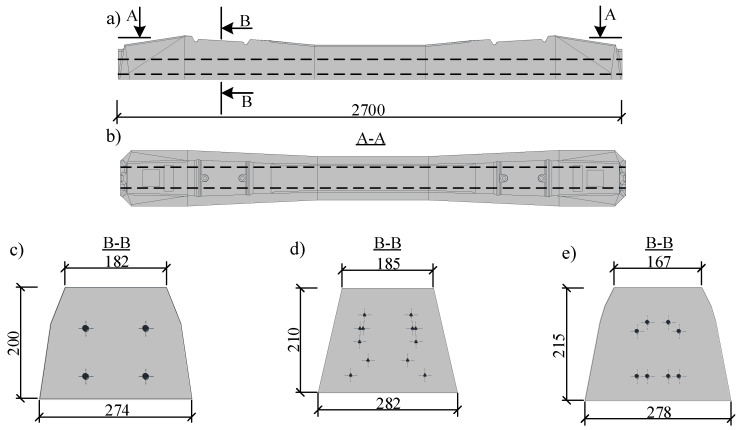
Rail seat section of sleepers: (**a**) side view; (**b**) top view; (**c**) Type I; (**d**) Type II; (**e**) Type III.

**Figure 2:**
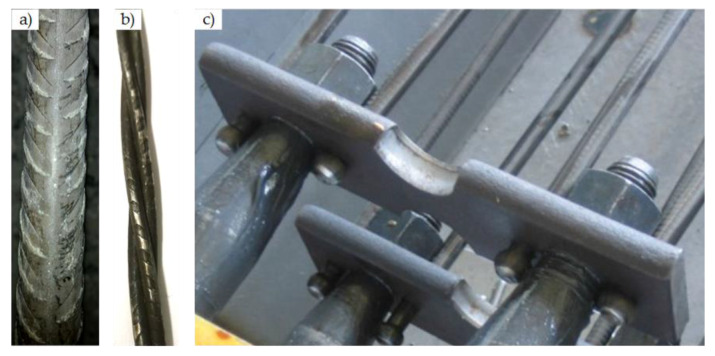
Reinforcement used in prestressed concrete sleepers: (**a**) Type I; (**b**) Type II; (**c**) Type III [6].

Three types (Type I, Type II, and Type III) of monoblock prestressed concrete sleepers were tested. Type I sleepers were reinforced with four prestressed indented steel bars (Figure 1c and Figure 2a). Type I sleepers were divided into two series: one series was reinforced with Ø9.6 mm prestressed steel bars and the other series–with Ø10.5 mm prestressed steel bars. Type II sleepers were reinforced with 12 prestressed indented three-wire steel strands of Ø6.8 mm nominal diameter (Figure 1d and Figure 2b). Type III sleepers were reinforced with eight prestressed plain steel bars of 7.0 mm diameter (Figure 1e and Figure 2c). The prestressing force in Type I and Type II sleepers was transferred to concrete through bond between reinforcement and concrete. In Type III sleepers, prestressing force was transferred to concrete through the anchorage plates installed at the end of sleeper (Figure 2c). Only prestressed longitudinal reinforcement was used in all types of sleepers and there were no non-prestressed bars or shear reinforcement. Additionally, the position of each prestressed reinforcement through the length of the sleeper was constant and spanning from one end of the sleeper to the other.

**Figure 3:**
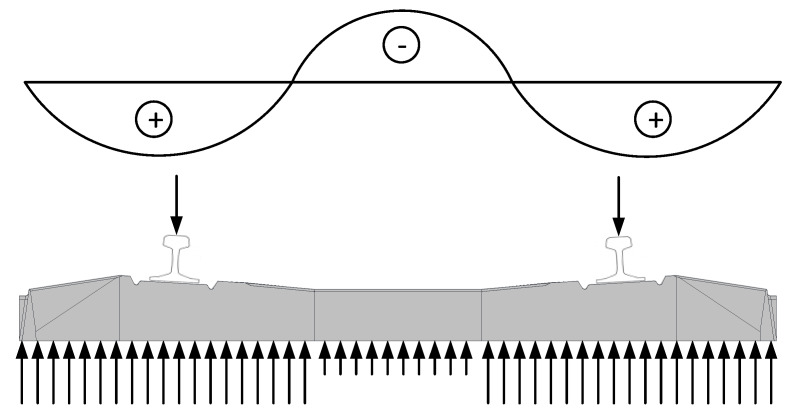
Bending moment diagram and stress distribution of the sleeper.

Usually, sleepers are affected by positive bending moment at the rail seat section and can be affected by negative bending moment in the middle of the sleeper (Figure 3). Therefore, the reinforcement is distributed through the height of cross-section. Additionally, the cross-section changes through the length of sleeper. It is higher and wider at the rail seat section and smaller and narrower in the middle of the sleeper (Figure 1a,b). The reinforcement in prestressed concrete sleepers is always straight through all the length of the element despite the method of production of sleepers. Therefore, the eccentricity of prestressed reinforcement regarding center of gravity of concrete cross-section varies at different sections of sleeper. It helps to resist positive bending moment at the rail seat section and negative bending moment in the middle of sleeper (Figure 3).

28 monoblock prestressed concrete sleepers were tested during experimental research (Table 1). Static and dynamic load tests were performed. Fourteen sleepers were tested under static load and 14 were also tested under dynamic load.

Main geometrical properties of each type of sleeper are provided in Table 2. Tested sleepers were reinforced with different types of prestressed reinforcement. Additionally, initial pretension force and initial stresses in prestressed reinforcement were different (Table 2). The highest pretension force was in Type III sleepers. However, the highest initial stresses in prestressed reinforcement were in Type II sleepers. Geometrical properties of Type I sleepers were the same. Part of the sleepers was prestressed with Ø9.6 mm indented bars and part–with Ø10.5 mm indented bars. Therefore, only reinforcement ratio and initial stresses in reinforcement were different. Geometrical properties of other types of sleepers differed. The eccentricity of reinforcement regarding center of gravity of cross-section was positive only in Type III sleepers. In other types of sleepers, the eccentricity of reinforcement was negative. It means that center of gravity of prestressed reinforcement is higher than center of gravity of rail seat section. This is an unfavourable property regarding cracking moment and stiffness of sleeper.

The highest utilization of reinforcement strength due to initial prestress is in Type I sleepers reinforced with Ø9.6 mm indented bars (σ_0_/f_pk_ = 0.79) and Type III sleepers (σ_0_/f_pk_ = 0.78). Contrary, the lowest utilization of reinforcement strength is in Type I sleepers reinforced with Ø10.5 mm indented bars (σ_0_/f_pk_ = 0.66) (Table 2).

### 2.2. Material Properties

The design concrete class of all tested sleepers was C50/60. Additionally, concrete cubes (100 × 100 × 100 mm) were cut from Type I, Type II, and Type III sleepers and standard concrete cubic (150 × 150 × 150 mm) strength was determined for each type of sleepers. Type I sleepers were reinforced with Ø9.6 mm or Ø10.5 mm prestressed indented steel bars. Type II and Type III sleepers were reinforced with Ø6.8 mm prestressed indented three-wire steel strands and with Ø7.0 mm prestressed plain steel bars respectively. The results of concrete compressive strength (f_c_), reinforcement strength (f_pk_), and reinforcement modulus of elasticity (E_p_) are provided in Table 3.

### 2.3. Experimental Test Setup

Experimental research of monoblock prestressed concrete sleepers was performed according to [20,21] which provides tests of prestressed concrete sleepers. An object of the research was the rail seat of the sleeper, therefore serviceability of sleeperd was monitored during static and dynamic load tests at the rail seat section. Pulsating and increasing load is applied to the concrete sleepers during dynamic bending test to simulate the situation in the track of effects of cyclic loads [20,21].

Crack width, strains at the surface of concrete and slip of reinforcement at the bottom of tension zone of cross-section were measured at the rail seat section of a sleeper during the static and dynamic load tests.

**Figure 4:**
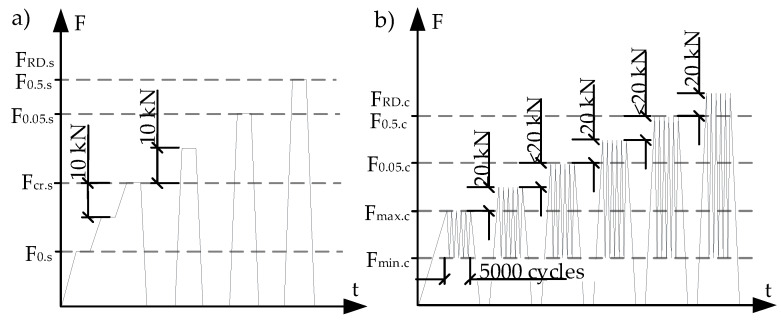
Test procedure: (**a**) under static load, (**b**) under dynamic load.

The first step of static load test (Figure 4a) was performed increasing applied load at a constant speed of 2 kN/s up to initial reference test load (F_0,s_). Furthermore, the load was increased in steps every 10 kN until cracking load (F_cr.s_). After the first crack appeared sleeper was unloaded at a constant speed of 2 kN/s. After that static load test was performed in steps. After every load step sleeper was unloaded. In each load step, static load was increased by 10 kN (Figure 4a). The static load test was thus performed until the failure of the sleeper.

The dynamic load test of the sleeper is divided into stages (Figure 4b). In every stage, sleeper is affected by 5000 load cycles. The dynamic load is of sinusoidal type with a frequency of 5 Hz. The dynamic load test starts with increasing static load at a constant speed of 2 kN/s until the initial reference test load (F_0,s_). This load is an upper value (F_max.c_) of the first stage of dynamic load. When F_max.c_ is reached sleeper is unloaded at a constant speed of 2 kN/s until the lower value (F_min.c_) of dynamic load. Then the dynamic load test begins. In every next load stage, the dynamic load is increased in steps every 20 kN. After every dynamic load stage, the sleeper is unloaded. During the test, lower value of dynamic load was kept constant in every stage and was equal to F_min.c_ = 50 kN. In the beginning and at the end of every load step crack widths and strains through the height of the cross-section were measured.

**Figure 5:**
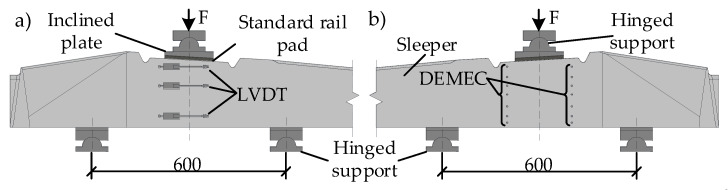
Test arrangement of the rail seat section for static and dynamic load: (**a**) strain measurement with LVDT sensors; (**b**) with DEMEC method.

Loading and supporting conditions were taken according to [21] and are provided in Figure 4. During static (Figure 4a) and dynamic (Figure 4b) load tests load was transferred through steel plates and standard rubber pad to the center part of the rail seat section (Figure 5). Crack width under load, residual crack width, strains on concrete surface through the height of cross-section, and slip of reinforcement in tension zone were measured during static and dynamic load tests (Figure 4). According to [20] crack width was measured 15 mm from the tension zone of the rail seat section with an optical microscope with an accuracy of 0.01 mm. The slip of reinforcement in all tested sleepers was measured with linear variable differential transducers (LVDT) with an accuracy of 0.001 mm.

**Figure 6:**
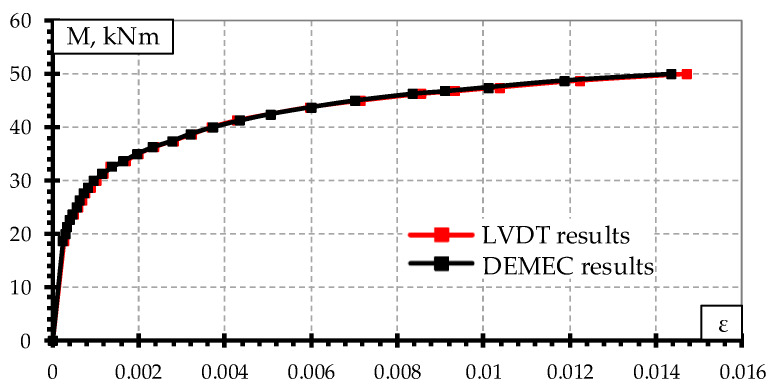
Results of concrete strains at tension zone of sleeper measured by two techniques.

Two techniques of measuring strains on the concrete surface through the height of the rail seat section during static and dynamic load tests were applied. On one side of sleeper concrete strains were measured with LVDT with an accuracy of 0.001 mm. Strains were measured at the level of mostly tensioned reinforcement, near the compressive zone and one measurement was performed at an intermediate level (Figure 5a). On the other side of the rail seat section, strains were measured with digital meter DEMEC (Figure 5b). Strains were measured in tension zone, at the level of every reinforcement and near compressive zone. The comparison of the strain results measured by two techniques are provided in Figure 6. The difference in results between two measurement techniques was 0–10%. The highest difference was near the failure load of the sleeper and difference in strains decrease with the decrease of acting load. Therefore, results show that both strain measuring techniques are accurate enough.

## 3. Results and Discussion

### 3.1. Static Load

#### 3.1.1. Cracking Moment

Type I and Type III sleepers need to resist higher design cracking moment compared to Type II sleepers (Table 4). Type II and Type III sleepers met design cracking moment requirements. However, Type I sleepers failed to satisfy design cracking moment.

Average cracking moment of Type I sleepers prestressed with Ø9.6 mm indented bars was the lowest compared to other sleepers (Table 4). This is conditioned by the highest negative eccentricity of prestressed reinforcement regarding centroid of cross-section and the lowest precompression force acting in cross-section (Table 2). Therefore, the stiffness of this type of sleeper was the lowest. All geometrical and material mechanical properties are the same for Type I sleepers except for the amount of reinforcement. Therefore, the increase of average cracking moment of sleepers reinforced with Ø10.5 mm indented bars by 49% can be influenced by the 20% increase of reinforcement ratio.

The stiffness of Type II sleepers was higher due to 5% higher precompression force in rail seat section and 5% lower negative eccentricity of prestressed reinforcement compared to Type I sleepers. However, the reinforcement ratio of sleepers prestressed with Ø10.5 mm indented bars was by 28% higher and concrete compressive strength was by 26% higher compared to Type II sleepers. Therefore, average cracking moment of Type II sleepers was slightly (2%) higher compared to sleepers reinforced with Ø10.5 mm indented bars. Additionally, the highest average cracking moment was determined for Type III sleepers. This was influenced by the highest precompression force in rail seat section and positive eccentricity of prestressed reinforcement. Therefore, Type III sleepers have the highest resistance to cracking.

#### 3.1.2. Bearing Capacity

**Figure 7:**
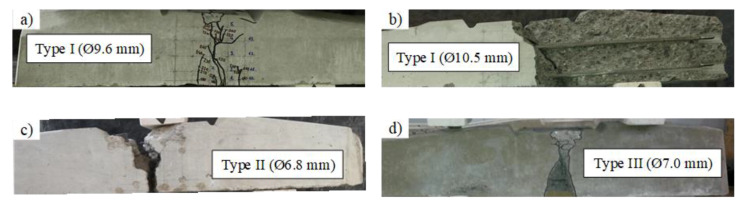
Types of failure of prestressed concrete sleepers under static load: (**a**) Type I (Ø9.6 mm); (**b**) Type I (Ø10.5 mm); (**c**) Type II; (**d**) Type III.

The experimental and design bearing capacities of all types of sleepers are provided in Table 5. Additionally, types of failure of different types of sleepers are provided in Table 6 and Figure 7. It can be seen that experimental bearing capacity of Type I sleepers was lower compared to the required design bearing capacity. The experimental bearing capacity of Type II and Type III sleepers met requirements for design bearing capacity. Despite lower design bearing capacity of Type II sleepers experimental bearing capacity almost met requirements for Type I and Type II sleepers. Therefore, with some small design corrections (e.g., additional strands) it could resist higher design moments.

From Table 5, it can be seen that bearing capacity under static load is similar for Type I and Type II sleepers. However, Type I sleepers reinforced with Ø9.6 mm bars failed due to rupture of reinforcement (Table 6). Despite the increase of diameter of reinforcement up to 10.5 mm and increase of cross-sectional area of reinforcement in Type I sleepers, the bearing capacity did not increase. It is related to different type of failure which was induced by slip of reinforcement and longitudinal splitting of concrete. The reason for that was insufficient anchorage length for bigger diameter (Ø10.5 mm) indented bars. Therefore, the strength of the bars was not completely utilized. The initial utilization of Ø10.5 mm indented bars was 66% due to initial prestress and initial utilization of Ø9.6 mm indented bars and Ø6.8 mm three-wire strands were 79% and 73% respectively (Table 2). The bearing capacity of Type III sleepers was by 18–23% higher compared to other types of sleepers. It is related to 2–5% higher area and 2–8% higher height of rail seat section compared to other types of sleepers. Additionally, total prestressed reinforcement ratio of Type III sleepers was by 6–10% higher compared to sleepers reinforced with Ø9.6 mm indented bars and Ø6.8 mm three-wire strands. However, it was by 13% lower compared to sleepers reinforced with Ø10.5 mm indented bars.

The reinforcement ratio and concrete compressive strength in Type II sleepers were by 6% and 26% lower respectively compared to sleepers reinforced with Ø9.6 mm bars. However, the strength of reinforcement in Type II sleepers was by 18% higher. Therefore, in total bearing capacity of Type II sleepers and sleepers reinforced with Ø9.6 mm bars was approximately equal (Table 5).

#### 3.1.3. Cracking and Deformations

**Figure 8:**
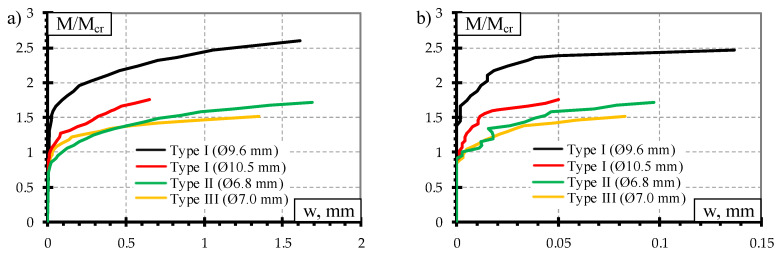
Comparison of crack width: (**a**) under the influence static load; (**b**) residual crack width.

**Figure 9:**
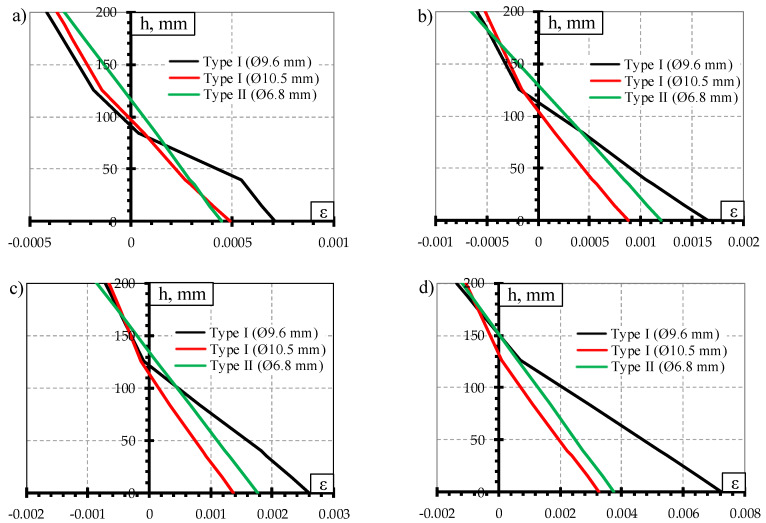
Comparison of strains through the height of rail seat section under static load: (**a**) M = 25 kNm; (**b**) M = 30 kNm; (**c**) M = 32.5 kNm; (**d**) M = 38.8 kNm.

The comparison of crack width under static load test is provided in Figure 8. The ratio between acting bending moment and cracking moment (M/M_cr_) was used for the comparison. It was done to evaluate differences in geometrical parameters, initial prestress force (Table 2), losses of prestress, and material mechanical properties (Table 3) between different types of prestressed concrete sleepers. Additionally, comparison of concrete strains through the height of the rail seat section under different bending moments is provided in Figure 9.

From Figure 8 it is clear that from all types of tested sleepers Type I sleepers reinforced with Ø10.5 mm bars had the lowest average crack widths and residual crack widths. The same trend can be seen in Figure 9 where strains at different values of bending moments were the lowest in tension zone of the rail seat section. As all other initial parameters of Type I sleepers were the same, the increase of crack width is conditioned by higher reinforcement ratio (Table 2). Additionally, the lowest average crack widths are related to failure due to slip of reinforcement and longitudinal splitting of concrete (Table 6). Therefore, the strength of reinforcement was not utilized due to insufficient anchorage length of reinforcement and propagation of cracking of sleepers was not able to develop further. However, the increase of bar diameter from 9.6 mm up to 10.5 mm in Type I sleepers conditioned up to 50% decrease of crack widths.

Type II and Type III sleepers had similar cracking behavior under static load (Figure 8). However, 5% lower second moment of inertia and 7% lower reinforcement ratio of Type II sleepers conditioned significantly lower crack width and residual crack width compared to Type III sleepers. Furthermore, with increasing load level, the difference between crack widths of Type II and Type III sleepers decreases. This phenomenon is conditioned by bond and anchorage properties of different prestressed reinforcement. Type II sleepers are prestressed with three-wire indented strands. The helical shape and geometrical imperfections of the strand increase roughness of reinforcement and increase bond between reinforcement and concrete [11,12]. Additionally, indentations on each wire of the strand also increase bond strength. Therefore, these properties restrict crack development at the place of the crack. These bond parameters together with Hoyer (wedge) effect at the ends of Type II sleepers ensure the required anchorage of reinforcement and restrict slip of reinforcement. Also, anchorage of reinforcement in Type III sleepers was ensured by special steel plates holding prestressed plain bars at the ends of sleepers. Additionally, a small portion of anchorage is ensured by bond between plain bars and concrete. However, plain bars have small bond resistance due to low roughness of the bars. Therefore, despite higher reinforcement ratio, restriction of crack development was smaller compared to Type II sleepers. Furthermore, the higher acting load on the Type III sleeper, the longer zone bonded between plain bar and concrete is disrupted. This means that the strains in reinforcement became higher due to distribution in longer distance. Therefore, from bending moments equal to 71% and 84% of bearing capacity of Type III and Type II sleepers respectively crack widths under the influence of static load was higher in case of Type III sleepers. As there was no rupture of plain bars during the failure of Type III sleeper reinforcement may not reach plastic strains and worked in an elastic range. Therefore, the type of anchorage of plain bars allowed low residual strains in the cracked zone.

The highest difference between experimental bearing capacity and experimental cracking moment (M_Rd,Exp_-M_cr,Exp_) was in case of Type III sleepers and Type I sleepers reinforced with Ø9.6 mm bars. It was 25–31% higher compared to other types of sleepers. Therefore, cracking development stage was longer in Type III and Type I sleepers reinforced with Ø9.6 mm bars. Up to bending moment equal to 67% and 77% of bearing capacity of Type III sleepers and Type I sleepers reinforced with Ø9.6 mm bars respectively the difference between average residual crack widths was 0–20%. However, under the influence of higher bending moments, the residual crack widths of Type I sleepers reinforced with Ø9.6 mm bars were propagating much faster and the difference increased rapidly. Additionally, the stiffness of these sleepers also decreased much faster. It is influenced by a higher reserve of bearing capacity of Type III sleepers which in turn depend on the higher height and area of the rail seat section.

However, the crack widths of Type III sleepers under the influence of static load were 16–60% lower compared to Type I sleepers reinforced with Ø9.6 mm bars. This difference was determined up to bending moments equal to 69% and 82% of bearing capacity of Type III sleepers and Type I sleepers reinforced with Ø9.6 mm bars, respectively. In this case, the reinforcement ratio differs only by 1%. Therefore, the lower crack width in Type III sleepers can be explained by 22% higher second moment of inertia which is conditioned by higher height and area of the rail seat section. Under the higher bending moments, crack widths under the influence of static load were higher in the case of Type III sleepers. This is influenced by longer zone of disrupted bond of plain bars along the sleeper which in turn conditioned higher strains at the cracked zone.

Average crack widths and average residual crack widths of Type II sleepers were higher compared to Type I sleepers reinforced with Ø9.6 mm bars. However, strains in the tension zone of the rail seat section of sleepers reinforced with Ø9.6 mm bars were higher (Figure 9). These contradictory results can be explained by the number of cracks that appeared during the static load test. All Type II sleepers had one crack propagating through the rail seat section up to the failure. Comparatively, sleepers reinforced with Ø9.6 mm bars had two or three cracks at the initial load stages. At higher load levels, one main crack was propagating through the rail seat section and others stopped. This was also the reason why the difference between average crack widths was decreasing with the increase of static load level.

**Figure 10:**
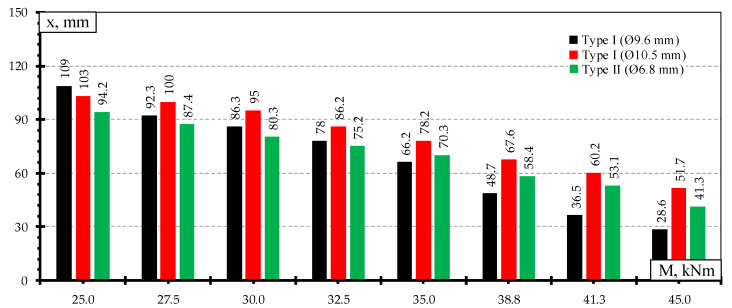
Comparison of the height of concrete compressive zone under static load.

The heights of the compression zone of the rail seat section of different types of sleepers under the influence of different levels of bending moments induced by static loads are provided in Figure 10. The lowest decrease of the height of concrete compression zone of the rail seat section was in Type I sleepers reinforced with Ø10.5 mm bars which had the lowest reinforcement ratio. Additionally, concrete strains in the tension zone of the rail seat section were the smallest (Figure 9). Therefore, it had the highest reserve of bearing capacity. However, bearing capacity of the rail seat section was not completely used due to type of failure (slip of reinforcement and longitudinal splitting of concrete along sleeper).

The highest decrease of the height of concrete compression zone was in Type I sleepers reinforced with Ø9.6 mm bars. The concrete strains in the tension zone of the rail seat section of this type of sleepers were also the highest (Figure 9). These differences also reflected on the lowest bearing capacity of sleepers reinforced with Ø9.6 mm bars (Table 5).

Approximately up to bending moment level of 63% of bearing capacity of Type II sleepers the height of concrete compression zone of this type of sleepers was lower. However, the decrease of the height of concrete compression zone of Type II sleepers was smaller under higher bending moments. Therefore, the height of concrete compression zone of Type II sleepers was higher compared to sleepers reinforced with Ø9.6 mm bars. It was influenced by 18% higher strength and by 8% lower initial utilization of three-wire strands compared to Ø9.6 mm bars.

### 3.2. Dynamic Load

#### 3.2.1. Bearing Capacity

The results of bearing capacity of different types of sleepers after dynamic load test are provided in Table 7. Type I sleepers did not meet design bearing capacity requirements by 3–7%. Type II and Type III sleepers satisfied design bearing capacity requirements. Additionally, as in case of static load test, bearing capacity of Type II sleepers was only about 1–2% lower compared to design bearing capacity requirements for Type I and Type III sleepers.

**Figure 11:**
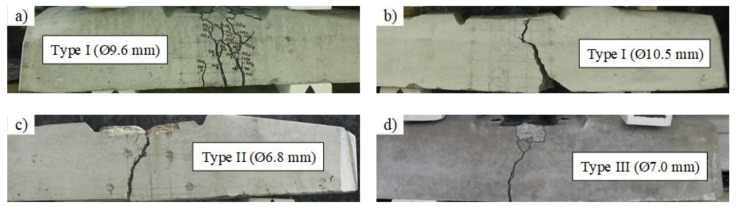
Type of failure of prestressed concrete sleepers under dynamic load: (**a**) Type I (Ø9.6 mm); (**b**) Type I (Ø10.5 mm); (**c**) Type II; (**d**) Type III.

Bearing capacity under dynamic load of Type I sleepers reinforced with Ø10.5 mm bars was slightly higher (4%) compared to sleepers reinforced with Ø9.6 mm bars. Types of failure for these sleepers were the same as in the case of static load (Table 6 and Table 8 and Figure 7 and Figure 11). Therefore, the strength of Ø10.5 mm bars was not completely utilized due to insufficient anchorage length (see also Section 3.1.2).

Bearing capacity of Type II sleepers was by 2–6% higher compared to Type I sleepers after the influence of dynamic load. The highest bearing capacity was of Type III sleepers and it was by 4–10% higher compared to other types of sleepers.

In all types of sleepers, dynamic load had negative impact on bearing capacity. Therefore, bearing capacity of sleepers reinforced with Ø9.6 mm indented bars, Ø10.5 mm indented bars, Ø6.8 mm three-wire strands and Ø7.0 mm plain bars decreased by 15%, 9%, 10%, and 21% respectively compared to static bearing capacity. The experimental results showed that the highest negative impact of dynamic load was on bearing capacity of Type III sleepers. Therefore, the difference in bearing capacity between Type II and Type III sleepers under dynamic load was smaller compare to the static test results (Table 5 and Table 7).

#### 3.2.2. Cracking and Deformations

**Figure 12:**
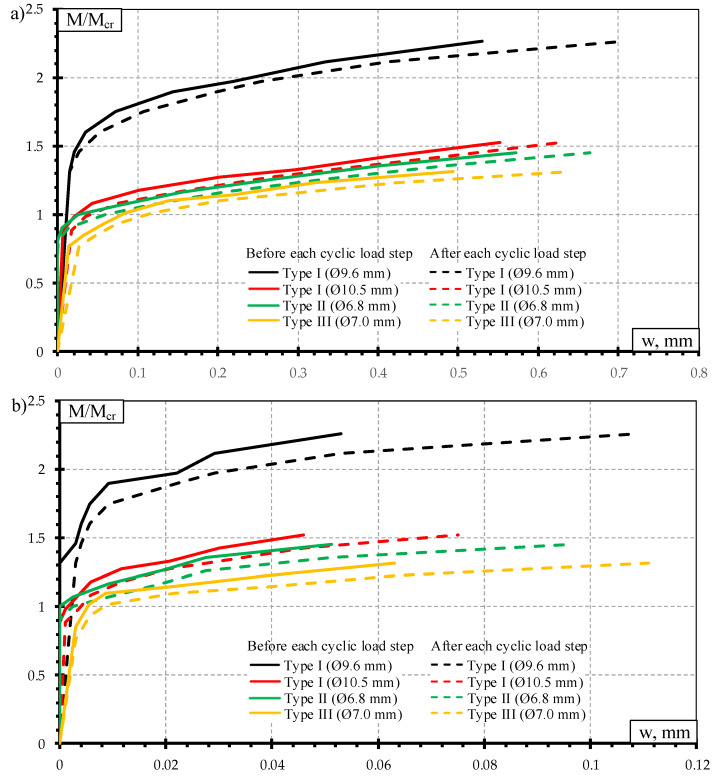
Comparison of crack width: (**a**) crack width under the influence of dynamic load; (**b**) residual crack width.

The crack width results of dynamic load test are provided in Figure 12. The development of crack widths is provided with respect to ratio between bending moment to cracking moment (M/M_cr_). The crack widths are provided at different stages of dynamic load test: crack widths before each dynamic load step and after each dynamic load step (Figure 12a); residual crack widths before each dynamic load step and after each dynamic load step (Figure 12b). At each dynamic load step, the crack width and residual crack width increased after the influence of dynamic load.

The highest bending moment and cracking moment ratio was in the case of Type I sleepers reinforced with Ø9.6 mm bars (Figure 12). Therefore, this type of sleepers affected by dynamic load had the longest crack development stage of all tested sleepers.

Even though Type I sleepers reinforced with Ø10.5 mm bars failed due to slip of reinforcement and longitudinal splitting of concrete (Table 8), the crack propagation after the influence of dynamic load was similar to other types of sleepers (Figure 12a,b). Additionally, residual crack widths of sleepers reinforced with Ø10.5 mm bars were the lowest after the influence of dynamic load compared to other types of sleepers. It means that this type of sleeper had the highest remaining precompression force after the influence of dynamic load.

**Figure 13:**
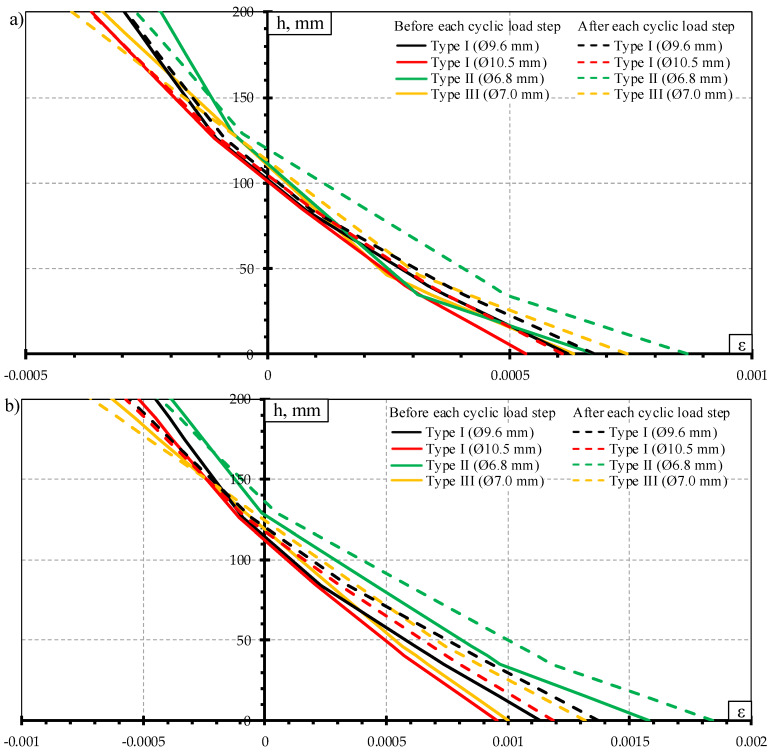

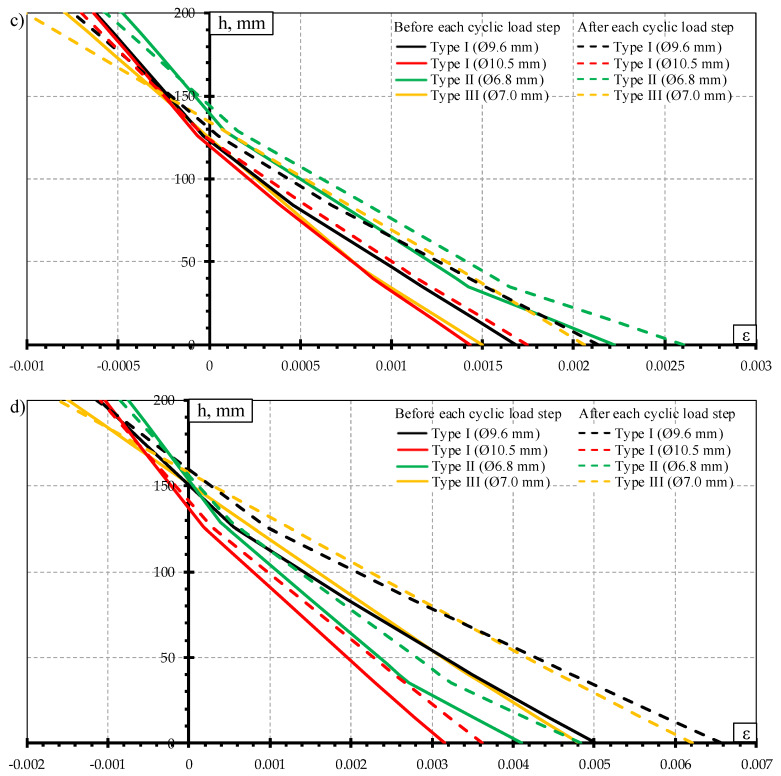
Comparison of strains through the height of rail seat section under dynamic load: (**a**) M = 25 kNm; (**b**) M = 30.3 kNm; (**c**) M = 32.5 kNm; (**d**) M = 38.8 kNm.

The highest average crack widths and residual crack widths up to bending moment level of 67% from bearing capacity were in Type II sleepers. Additionally, concrete strains in the tension zone of the rail seat section were also the highest in Type II sleepers up to the same bending moment level (Figure 13). At higher bending moment levels difference between crack widths of Type II sleepers and other types of sleepers decreased and crack widths of other types of sleepers became equal or higher compared to Type II sleepers.

The average crack widths and residual crack widths of Type III sleepers at the initial dynamic loads were higher than crack widths of Type I sleepers. However, from bending moment level of about 58% from bearing capacity of Type III sleepers crack widths became similar to sleepers reinforced with Ø9.6 mm bars. The difference was about 2–9%. Additionally, crack widths of sleepers reinforced with Ø10.5 mm bars became 2–26% higher compared to Type III sleepers. Type I sleepers are prestressed with indented bars which increase bond of reinforcement. On the other hand, the same indentations on the surface of bars cause additional stress concentration in surrounding concrete. Therefore, local splitting of concrete appears near indentations. Higher dynamic load levels, in turn, induce higher stress concentration near indentations. Therefore, constantly changing dynamic load level at higher frequency can induce severe splitting cracks and damage bond of reinforcement which in turn negatively affects crack width. Usually, the higher the diameter of reinforcement, the bigger the indentations on the surface of the bar and higher splitting stress around reinforcement. As Type III sleepers are reinforced with plain bars there is no splitting stress around reinforcement. Therefore, crack widths of sleepers reinforced with Ø10.5 mm bars were higher compared to Type III sleepers under the influence of higher dynamic load levels.

The concrete strains in the tension zone of the rail seat section of Type I sleepers reinforced with Ø10.5 mm bars were the lowest up to bending moment level of 80% from bearing capacity (Figure 13). However, concrete strains in the tension zone of Type I sleepers reinforced with Ø9.6 mm bars were higher even though average crack width under the influence of dynamic load was 12–34% lower compared to sleepers reinforced with Ø10.5 mm bars. It can be explained by a higher number of cracks in sleepers reinforced with Ø9.6 mm bars. Initially, several cracks appeared in rail seat section of sleepers reinforced with Ø9.6 mm bars and under higher bending moment levels some of the cracks stopped propagating and one or two main cracks developed further.

**Figure 14:**
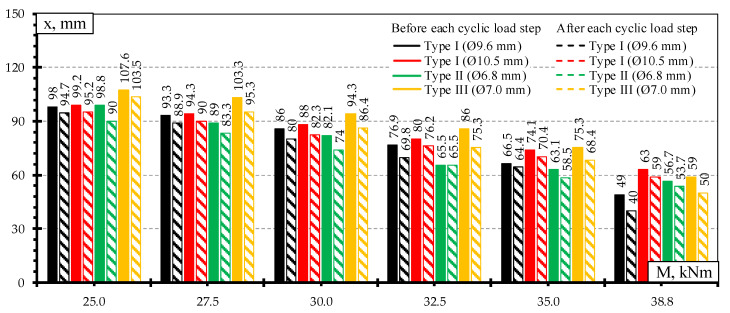
Comparison of the height of the concrete compressive zone before and after each dynamic load level.

The height of the concrete compression zone of the rail seat section of different types of sleepers under the influence of different levels of bending moments induced by dynamic load is provided in Figure 14. After each dynamic load step height of concrete compression zone decreased. The lower the height of the concrete compression zone of the rail seat section, the higher stresses in reinforcement in tension zone (Figure 13 and Figure 14). This results in accelerated development of crack width of rail seat section and reduction in sleeper stiffness under dynamic load. Therefore, dynamic load causes greater damage to sleepers and is more dangerous than static load. The highest decrease of the height of the concrete compression zone was in Type I sleepers reinforced with Ø9.6 mm bars.

**Figure 15:**
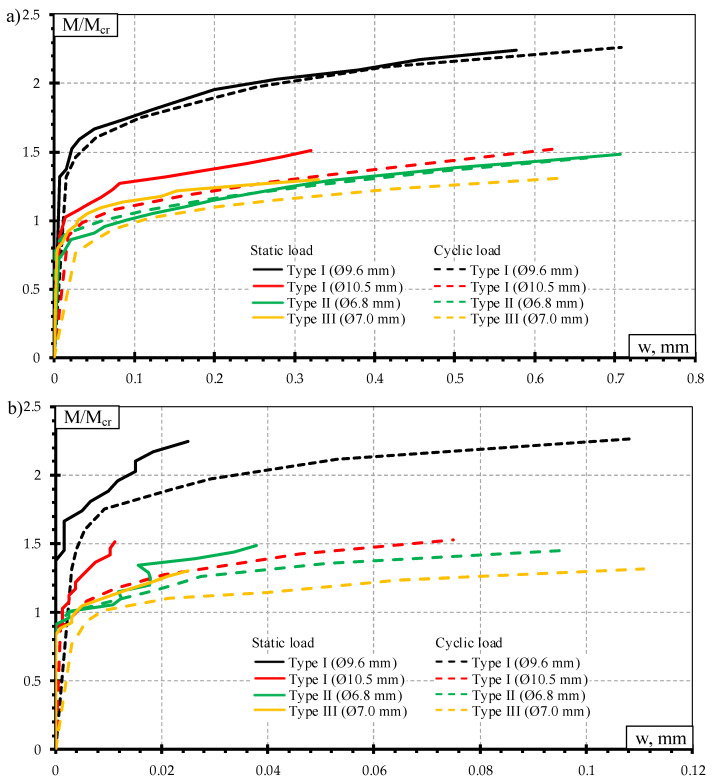
Comparison of crack width under static and dynamic load: (**a**) crack width under the influence of static and dynamic load; (**b**) residual crack width.

Comparison of crack widths induced by static and dynamic load is provided in Figure 15. The crack widths are compared up to the same bending moment level caused by static and dynamic load. Under the same load levels, crack widths induced by static load are significantly lower than in case of dynamic load. Additionally, for all types of sleepers, dynamic load conditioned higher increase of residual crack widths compared with residual crack widths induced by static load (Figure 15b). Therefore, dynamic load is more critical for the propagation of residual crack widths.

In dynamic load testing, average crack width values between each type of sleeper were more evenly distributed compared to static load test. Therefore, crack widths of Type I sleepers reinforced with Ø10.5 mm bars in case of dynamic load effect were similar to crack widths of other types of sleepers. It means that Type I sleepers reinforced with Ø10.5 mm bars were more susceptible to dynamic load effect. Therefore, dynamic load reduces stiffness of this type of sleepers faster than for other types of sleepers.

**Figure 16:**
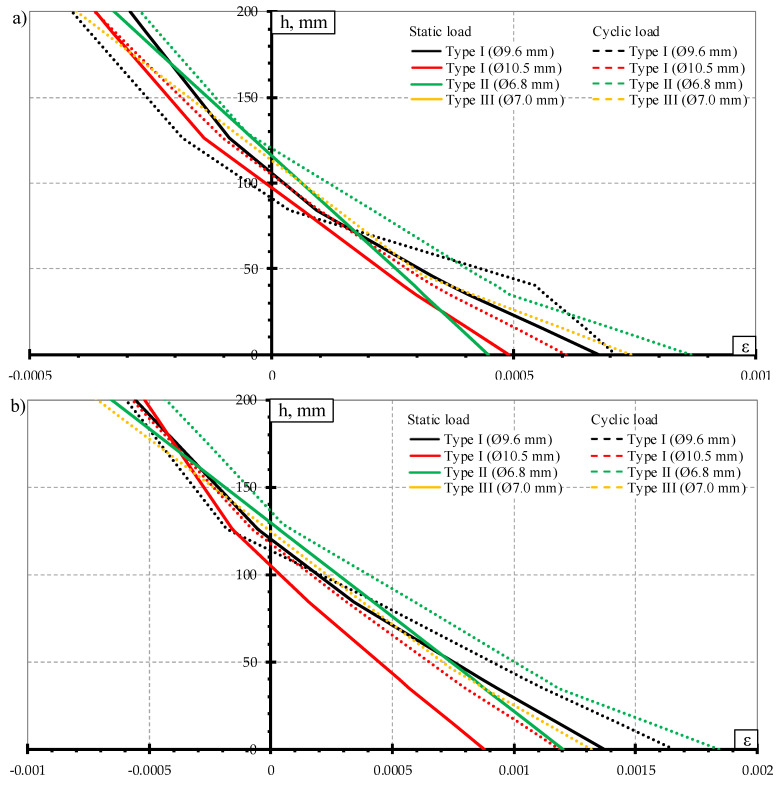

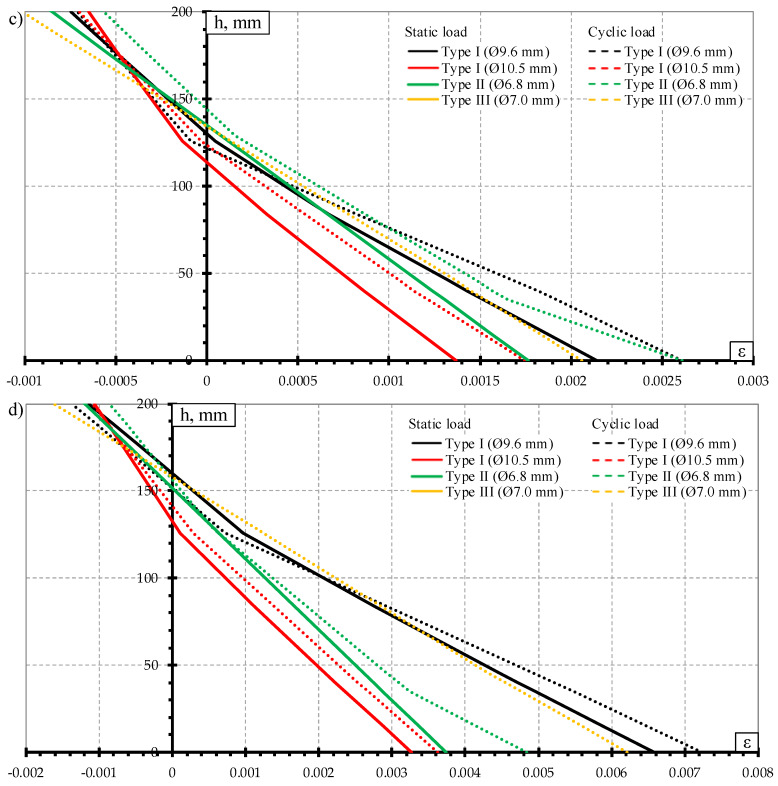
Comparison of concrete strains under static and dynamic load: (**a**) M = 25 kNm; (**b**) M = 30 kNm; (**c**) M = 32.5 kNm; (**d**) M = 38.8 kNm.

Furthermore, comparison of concrete strains through the height of the rail seat section under the influence of static and dynamic load showed the same trend as in case of crack widths comparison. The influence of dynamic load induced higher strains in the tension zone of the rail seat section (Figure 16). Therefore, dynamic load had an additional negative effect on the degradation of the rail seat section. Dynamic load induced stress concentration at the place of opened crack. Horizontal internal cracks appear perpendicular to the crack in the area of contact between reinforcement and concrete, where bond is damaged. Then, in the tensile zone of the rail seat section friction appears between crack plane and reinforcement and reinforcement is worn due to dynamic load (dynamic loading and unloading). Reinforcement bends at a slightly larger angle at the edges of concrete on the plane of the crack. The lower the stiffness of the sleeper, the greater the angle of bent reinforcement under dynamic load. The part of reinforcement in crack closest to the load line (center of the rail seat section) is more worn than reinforcement in uncracked part of the rail seat section.

**Figure 17:**
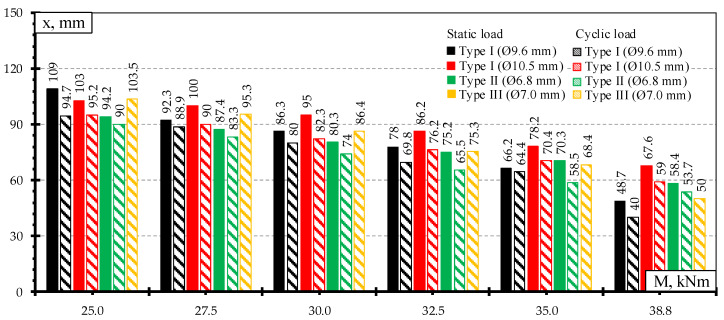
Comparison of the height of the concrete compression zone under the influence of static and dynamic load.

The comparison of the height of concrete compression zone of different types of sleepers tested under static and dynamic load is provided in Figure 17. It can be seen that the height of concrete compression zone was lower after the influence of dynamic load compared to static load test results. The height of the concrete compression zone is directly related to bearing capacity of flexural element. Therefore, bearing capacity of all types of sleepers decreased under the influence of dynamic load compared to static load (Table 5 and Table 7).

## 4. Conclusions

After analyzing the experimental results, it was found that slippage of reinforcing bars occurred at the end of Type I sleepers reinforced with Ø9.6 mm and Ø10.5 mm indented bars during the test. This indicates that the diameter of reinforcement (reinforcement) was selected incorrectly and anchorage length of prestressed bars was insufficient. Conversely, in Type II sleepers reinforced with three-wire strands, the bond between reinforcement and concrete was adequate and strands did not slip during the test. Additionally, the anchorage and consequently bond of plain bars were ensured by additional anchor plates at the ends of Type III sleepers.

The research results showed that experimental cracking and failure moments of Type I sleepers were lower than design moments. This means that the structural solution of these type of sleepers was not suitable, because their behavior did not correspond to design evaluation of sleepers. The research has shown that failure occurred due to insufficient anchorage of reinforcement in concrete. In the case of Type II and Type III sleepers, the experimental values of cracking and failure moments were higher than the design ones, because anchorage of reinforcement in concrete was sufficient.

The research showed that reinforcement and rail seat section of Type III sleepers are chosen irrationally. During static and dynamic load tests, the sleepers failed in the concrete compression zone. This indicates that sleeper reinforcement does not match the selected cross-section of sleeper.

The use of higher diameter (Ø10.5 mm) prestressed bars improved the Type I sleeper’s resistance to cracking at rail seat section. Therefore, propagation of residual crack width was slower compared to sleepers reinforced with lower diameter (Ø9.6 mm) prestressed bars. However, a higher reinforcement ratio negatively influenced anchorage zone of sleeper and sleepers failed due to slip of reinforcement accompanied by longitudinal splitting of concrete. Therefore, confining reinforcement or anchors could be introduced at the ends of the sleeper to minimize the concrete splitting effect.

The distribution of crack width between each type of sleepers was more even under the influence of dynamic load compared to static load. Therefore, in contrast to static load test, crack widths of Type I sleepers reinforced with Ø10.5 mm bars were similar to crack widths of other types of sleepers under the influence of dynamic load. It means that Type I sleepers reinforced with Ø10.5 mm bars are more susceptible to dynamic load effect. Therefore, dynamic load reduces stiffness of this type of sleepers faster than for other types of sleepers.

It was found that the bearing capacity of Type I sleepers reinforced with Ø9.6 mm and Ø10.5 mm indented bars, Type II and Type III sleepers after the influence of dynamic load decreased by 15%, 9%, 10%, and 21% respectively compared with static test results. The greater degradation of prestressed concrete sleepers under the influence of dynamic load is related to higher deformation of reinforcement in the crack plane and greater damage on bond between reinforcement and concrete near the crack plane. Additionally, the character of failure of each type (Type I, Type II, and Type III) of the sleeper was the same after static and dynamic load tests.

## Figures and Tables

**Table 1:** Experimental program.

Type of Load	Type I		Type II	Type III
	Ø9.6 mm	Ø 10.5 mm	Ø6.8 mm	Ø7.0 mm
Static load	2	3	3	6
Dynamic load	2	3	3	6

**Table 2:** Initial parameters.

Parameter	Type I		Type II	Type III
Ø_p_ (mm)	Ø9.6	Ø10.5	Ø6.8	Ø7.0
Type of reinforcement	Indented bar	Indented bar	Indented three-wire strand	Plain bar
n_p_ (pcs.)	4	4	12	8
P_0_ (kN)	360.8	360.8	380	400
σ_0_ (MPa)	1247	1042	1355	1300
σ_0_/f_pk_	0.79	0.66	0.73	0.78
A_c_, mm^2^	47,593	47,593	49,035	50,123.5
e_p_, mm	−9.3	−9.3	−5	6.9
ρ_p_	0.00608	0.00733	0.00572	0.00614

A_c_—an area of concrete cross-section; e_p_—eccentricity of prestressed reinforcement; ρ_p_—prestressed reinforcement ratio.

**Table 3:** Material properties.

Parameter	Type I		Type II	Type III
	Ø9.6 mm	Ø10.5 mm	Ø6.8 mm	Ø7.0 mm
Properties of reinforcement				
f_pk_ (MPa)	1570	1570	1860	1670
E_p_ (GPa)	200	200	200	200
	Properties of concrete			
f_c_ (MPa)	108.4	101.2	80.6	85.3

**Table 4:** Cracking moments of prestressed concrete sleepers.

Cracking Moment	Type I		Type II	Type III
	Ø9.6 mm	Ø10.5 mm	Ø6.8 mm	Ø7.0 mm
M_cr,Exp_, kNm	15.2	22.6	23.1	25.3
M_cr,Design_, kNm	22.7	22.7	18.7	22.8

**Table 5:** Bearing capacity of prestressed concrete sleepers after the influence of static load.

Bearing Capacity	Type I		Type II	Type III
	Ø9.6 mm	Ø10.5 mm	Ø6.8 mm	Ø7.0 mm
M_Rd,Exp_, kNm	55.2	53.2	55.0	65.4
M_Rd,Design_, kNm	56.8	56.8	46.8	57.0

**Table 6:** Types of failure of sleepers after the influence of static load.

Type of Sleeper	Type of Failure
Type I Ø9.6 mm	Rupture of reinforcement
Type I Ø10.5 mm	Slip of reinforcement and longitudinal splitting of concrete
Type II Ø6.8 mm	Rupture of reinforcement
Type III Ø7.0 mm	Failure of concrete compression zone

**Table 7:** Bearing capacity of prestressed concrete sleepers after the influence of dynamic load.

Bearing Capacity	Type I		Type II	Type III
	Ø9.6 mm	Ø10.5 mm	Ø6.8 mm	Ø7.0 mm
M_Rd,Exp_, kNm	46.7	48.5	49.3	51.5
M_Rd,Design_, kNm	50.0	50.0	41.1	50.2

**Table 8:** Types of failure of sleepers after the influence of dynamic load.

Type of Sleeper	Type of Failure
Type I Ø9.6 mm	Slip of reinforcement and longitudinal splitting of concrete or rupture of reinforcement
Type I Ø10.5 mm	Slip of reinforcement and longitudinal splitting of concrete
Type II Ø6.8 mm	Rupture of reinforcement
Type III Ø7.0 mm	Failure of concrete compression zone
